# Characterization of the immunomodulatory properties of alveolar bone-derived mesenchymal stem cells

**DOI:** 10.1186/s13287-020-01605-x

**Published:** 2020-03-05

**Authors:** Chen Cao, Susan Tarlé, Darnell Kaigler

**Affiliations:** 1 Department of Periodontics and Oral Medicine, School of Dentistry, University of Michigan, 1011 N. University, Ann Arbor, MI 48109 USA; 2 Department of Biomedical Engineering, College of Engineering, University of Michigan, 1011 N. University, Ann Arbor, MI 48109 USA

**Keywords:** Stem cells, Mesenchymal stem cell, Cell therapy, IL-6, MCP-1, Immunomodulation

## Abstract

**Background:**

Recently, mesenchymal stem cells (MSCs) have been shown to have immunomodulatory properties which hold promise for their clinical use to treat inflammatory conditions. Relative to bone marrow-derived MSCs (BMSCs), which are typically isolated from the iliac crest, we have recently demonstrated that MSCs can be predictably isolated from the alveolar bone (aBMSCs) by less invasive means. As such, the aim of this study was to characterize the immunomodulatory properties of aBMSCs relative to BMSCs.

**Methods:**

aBMSCs isolated from the human alveolar bone and BMSCs isolated from the human bone marrow of the iliac crest were cultured in the same conditions. Cytokine arrays and enzyme-linked immunosorbent assays (ELISA) of a conditioned medium were used to evaluate differences in the secretion of cytokines. In different functional assays, aBMSCs and BMSCs were cocultured with different types of immune cells including THP-1 monocytes, macrophages, and peripheral blood mononuclear cells (PBMCs) to evaluate their effects on important immune cell functions including proliferation, differentiation, and activation.

**Results:**

The protein arrays identified interleukin (IL)-6 and monocyte chemoattractant protein (MCP)-1 to be the major cytokines secreted by aBMSCs and BMSCs. ELISA determined that aBMSCs secreted 268.64 ± 46.96 pg/mL of IL-6 and 196.14 ± 97.31 pg/mL of MCP-1 per microgram of DNA, while BMSCs secreted 774.86 ± 414.29 pg/mL of IL-6 and 856.37 ± 433.03 pg/mL of MCP-1 per microgram of DNA. The results of the coculture studies showed that aBMSCs exhibited immunosuppressive effects on monocyte activation and T cell activation and proliferation similar to BMSCs. Both aBMSCs and BMSCs drove macrophages into an anti-inflammatory phenotype with increased phagocytic ability. Taken together, these data suggest that aBMSCs have potent immunomodulatory properties comparable to those of BMSCs.

**Conclusions:**

The findings of this study have important implications for the development of immunomodulatory stem cell therapies aimed to treat inflammatory conditions using aBMSCs, a more feasible tissue source of MSCs.

## Background

For many years, mesenchymal stem cells (MSCs) from the bone marrow have shown promise as a viable cell type for cell therapies due to their regenerative properties [1–6]. More recently, the therapeutic potential of MSCs has significantly increased in that recent evidence demonstrates these cells also have potent immunomodulatory properties and can be isolated from more readily accessible tissues other than the bone marrow, including the adipose tissue [7], umbilical cord [8, 9], placenta [10], and oral/dental tissues [11]. Compared to MSCs derived from the bone marrow of the iliac crest (BMSCs), our group and others have recently demonstrated that MSCs can be predictably isolated by less invasive means from the alveolar bone [12–14]. Alveolar bone-derived MSCs (aBMSCs) meet the current MSC “gold standards” [15], i.e., they are highly positive for MSC markers CD73, CD90, and CD105, negative for CD11b, CD19, and CD45; exhibit multipotent differentiation capacity into osteoblasts, adipocytes, and chondroblasts; and induce ectopic bone formation in vivo [12]. Additionally, preclinical animal models have shown the potential of aBMSCs in cell-mediated regeneration of bone defects [14, 16].

In contrast to the well-established regenerative properties of MSCs, the immunomodulatory functions of MSCs have only recently been studied [2, 4, 17]. Preclinical studies on MSCs’ immunomodulatory properties have identified the importance of MSC-secreted soluble factors (cytokines, chemokines, growth factors, etc.) and their interactions with other immune cells in the context of treating chronic inflammatory and autoimmune diseases (e.g., systemic lupus erythematosus/SLE), and transplant complications (e.g., graft-versus-host disease/GvHD) [2]. Many clinical trials using MSCs as cell therapies for such conditions have been conducted, and a number of them report promising results although challenges remain [17]. MSC injections have shown benefits in patients with steroid-refractory acute GvHD after allogeneic hematopoietic stem cell transplantation [18, 19]. In another cell therapy, the European Commission recently approved the first MSC pharmaceutical agent (Alofisel) to treat enterocutaneous fistulas developed in patients with Crohn’s disease, a chronic inflammatory bowel disease [17]. MSC therapy has also been recommended as a third-line treatment for acute steroid-refractory GvHD in the UK [18] and has been granted conditional approval for treatment of children with GvHD in other countries throughout the world [17].

Though aBMSCs and BMSCs are both isolated from the bone tissues, an important development difference exists between the two types of the bone from which these stem cells are derived. The craniofacial bones, including the alveolar bone, are derived from the ectoderm, more specifically, the neural crest cells; other bones, including the iliac crest bone, originate from embryonic mesodermal cells [20]. Hence, it cannot be assumed that aBMSCs have the same or comparable immunomodulatory functions as the BMSCs. As such, the aim of this study was to determine if aBMSCs exhibited immunomodulatory properties in that the immunomodulatory properties of aBMSCs have not been previously reported. It would be of high interest to test this hypothesis because aBMSCs may serve as an alternative to BMSCs to treat immune-mediated diseases, particularly since aBMSCs can be obtained more readily and more cost-effectively than BMSCs. In this study, we explored and characterized the immunomodulatory functions of aBMSCs relative to BMSCs through an assessment of cytokine and growth factor secretion and in vitro cell functional assays.

## Materials and methods

### Cell culture

Following the University of Michigan Institutional Review Board approval (IRB #HUM00034368), alveolar bone specimens were obtained from patients undergoing routine oral surgical procedures, and the bone marrow was collected from patients undergoing routine bone marrow aspirations from the iliac crest. BMSCs were isolated from the bone marrow aspirated from the iliac crest [21] while aBMSCs were isolated from alveolar bone samples as previously described [12]. Both aBMSCs and BMSCs were cultured in Minimum Essential Media α containing ribonucleosides and deoxyribonucleosides (MEM α; Thermo Fisher Scientific, Waltham, MA, USA) supplemented with 15% fetal bovine serum (FBS; MilliporeSigma, Burlington, MA, USA), 0.1 mM l-ascorbic acid-2-phosphate (MilliporeSigma), and 25 μg/mL gentamicin (Thermo Fisher Scientific). The isolated cells were confirmed to be MSCs by performing mesodermal differentiation assays (osteogenic, adipogenic, and chondrogenic) and immunophenotype characterization as described by Dominici et al. [15]. aBMSCs and BMSCs at passages 4–8 were used in this study.

Primary peripheral blood mononuclear cells (PBMCs) were obtained from BioIVT (Westbury, NY) and cultured in RPMI 1640 medium (Thermo Fisher Scientific) supplemented with 10% FBS, 50 U/mL penicillin, and 50 μg/mL streptomycin (Thermo Fisher Scientific). THP-1 monocytic cell line was obtained from the American Type Culture Collection (ATCC, Manassas, VA, USA) and maintained between 2 and 8 × 10^5^/mL in the RPMI-FBS medium described herein with the addition of 0.05 mM 2-mercaptoethanol (ATCC).

### Assessment of cytokine and growth factor secretion

aBMSCs and BMSCs were cultured to 75–80% confluent in T-25 flasks and then washed briefly with DPBS and incubated with 2.5 mL basal medium (MEM α without serum) for 24 h. The conditioned media (CM) were collected, centrifuged at 4 °C to remove cellular debris, and stored at − 80 °C until use. The secretion of cytokines and growth factors by aBMSCs and BMSCs was assessed by a Human Cytokine Array C3 (RayBiotech, Peachtree Corners, GA, USA; detecting 42 human cytokines: ENA-78, GCSF, GM-CSF, GRO, GRO-α, I-309, IL-1α, IL-1β, IL-2, IL-3, IL-4, IL-5, IL-6, IL-7, IL-8, IL-10, IL-12 p40/p70, IL-13, IL-15, IFN-γ, MCP-1, MCP-2, MCP-3, MCSF, MDC, MIG, MIP-1δ, RANTES, SCF, SDF-1, TARC, TGF-β1, TNF-α, TNF-β, EGF, IGF-I, Angiogenin, Oncostatin M, Thrombopoietin, VEGF-A, PDGF BB, Leptin). The concentrations of IL-6 and MCP-1 in CM were evaluated by ELISA kits (R&D Systems, Minneapolis, MN, USA).

### DNA isolation and quantification

Cells were lysed in passive lysis buffer (PLB; Promega, Madison, WI, USA)) and frozen at − 80 °C until processed as follows. Thawed cell lysates in PLB were sonicated and centrifuged at 10,000 rpm for 10 min at 4 °C. The pellet was resuspended and sonicated in Caron’s buffer and centrifuged at 13,000 × g for 10 min at 4 °C. The supernatant was collected, and the DNA concentration was quantified by Qubit Assay using a Qubit fluorometer (Thermo Fisher Scientific).

### Cytokine expression in THP-1 cells

2.0 × 10^5^ THP-1 cells were either cocultured with 1 × 10^5^ aBMSCs/BMSCs or cultured alone in 3 mL THP-1 growth medium in 6-well plates for 72 h. To evaluate the effect of aBMSCs/BMSCs through secreted soluble factors, aBMSCs or BMSCs were plated at 1 × 10^4^/cm^2^ for 16–18 h overnight and then incubated with THP-1 growth medium for 24 h to collect CM. The media were centrifuged to remove any cells and kept at − 80 °C until added to THP-1 cultures: 1 × 10^5^ THP-1 cells in 2 mL fresh THP-1 growth medium per well with additional 1 mL of aBMSCs/BMSCs CM. After 72 h of coculture or monoculture, 1 μg/mL lipopolysaccharide (LPS) from *Escherichia coli* (*E. coli*; MilliporeSigma) was added to the cell culture for additional 4 h of incubation in the presence of 5 μg/mL brefeldin A (to block secretion of cytokines; BioLegend, San Diego, CA, USA). The non-adherent cells were harvested and subjected to flow cytometry detecting intracellular tumor necrosis factor α (TNF-α) as previously described [22]. In brief, collected cells were first incubated with peridinin-chlorophyll-protein/cyanine5.5 (PerCP/Cy5.5)-conjugated anti-human CD90 antibody (Thermo Fisher Scientific) to label MSCs, if any, to be excluded from counting THP-1 cells, and then fixed and permeabilized using the Cytofix/Cytoper Fixation/Permeabilization kit from BD Biosciences (Hercules, CA, USA), followed by incubation with phycoerythrin (PE)-conjugated anti-human TNF-α antibody. Cells were rinsed, suspended in Cell Staining Buffer (BioLegend), and analyzed by Bio-Rad ZE5 Cell Analyzer (San Jose, CA, USA).

### Phagocytosis of *E. coli* in THP-1 macrophages

Similar to what has been previously described [23], 2 × 10^5^ THP-1 cells plated per well of 6-well plates were differentiated into macrophages with 10 ng/mL phorbol 12-myristate 13-acetate (PMA) for 96 h in the presence or absence of 1 × 10^5^ aBMSCs or BMSCs cultured in Transwell inserts (Corning Inc., Corning, NY, USA). For control purpose, 100 ng/mL of IL-4, an M2 inducer, was added to some wells of THP-1 cells cultured alone at 24 h since the beginning of PMA induction. At the end of differentiation incubation, the Transwell inserts were removed, and the bottom wells with THP-1 macrophages were briefly rinsed with PBS and then incubated with 10 μg/mL AlexaFluor (AF) 488-conjugated *E. coli* (Thermo Fisher Scientific) for 1 h. After quenching the extracellular fluorescence with 0.4% Trypan Blue, the THP-1 macrophages were washed three times, detached with 5 mM Na_2_ EDTA, and analyzed by Bio-Rad ZE5 Cell Analyzer.

### Immunosuppression on T lymphocyte response

T lymphocyte proliferation was studied in vitro as previously described [24]. Briefly, primary human peripheral blood mononuclear cells (PBMCs) were first labeled with 2 μM CFSE (formally known as 5-(and 6)-carboxyfluorescein diacetate succinimidyl ester), a cell-permeable fluorescent dye, and then cultured at 1 × 10^5^ or 2 × 10^5^ per well of a 96-well plate in the presence or absence of 1 × 10^4^ aBMSCs or BMSCs with or without ImmunoCult™ Human CD3/CD28/CD2 T Cell Activator (anti-CD3/CD28/CD2 antibody complexes; StemCell Technologies, Vancouver, BC, Canada) for 5 days. Non-adherent cells were harvested and subjected to flow cytometry using PE-conjugated anti-human CD4 and allophycocyanin (APC)-conjugated anti-human CD8 antibodies (BioLegend) to gate for CD4^+^ and CD8^+^ T lymphocytes. The culture media were collected, centrifuged, and stored at − 80 °C. The interferon γ (IFN-γ) levels in the supernatants were assessed by ELISA (BioLegend) as a measure of the T cell activation.

### Data analysis

The flow cytometry data were analyzed with FCS Express 6 and 7 (De Novo Software, Pasadena, CA, USA). The results in this study are presented as mean ± standard deviation (SD). The statistical analyses were performed in Prism 8 (GraphPad Software, San Diego, CA, USA) using an unpaired two-tailed *T* test. A difference with a *P* value less than 0.05 was considered statistically significant.

## Results

### Cytokine and growth factor secretion in aBMSCs and BMSCs

**Fig. 1:**
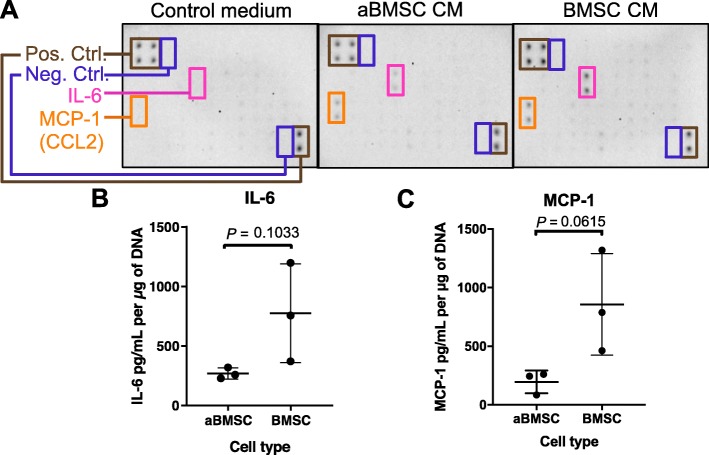
IL-6 and MCP-1 are the major cytokines secreted by resting aBMSCs and BMSCs. aBMSCs or BMSC-conditioned media were collected and analyzed by a protein array detecting human pro- and anti-inflammatory cytokines and growth factors (**a**), and ELISA kits detecting IL-6 (**b**) and MCP-1 (**c**). **b**, **c** The cytokine levels were normalized according to the DNA content of the cells. *n* = 3 for each cell type from three different donors

Since cytokines play important roles in the regulation of immune responses, we first collected conditioned medium (CM) of aBMSCs and BMSCs to determine their production of soluble cytokines. Among 42 pro- and anti-inflammatory cytokines and growth factors tested, only IL-6 and MCP-1 (CCL2) were found to be secreted by both aBMSCs and BMSC at detectable levels (Fig. 1a). Secretion levels of IL-6 and MCP-1 were quantified by ELISA and showed the following: aBMSCs and BMSCs secreted IL-6 at 268.64 ± 46.96 and 774.86 ± 414.29 pg/mL per μg of DNA, respectively (Fig. 1b), and MCP-1 at 196.14 ± 97.31 and 856.37 ± 433.03 pg/mL per μg of DNA, respectively (Fig. 1c). Overall, aBMSCs secreted less IL-6 and MCP-1 than BMSCs but this difference was not significant for either IL-6 (*P* = 0.1033; Fig. 1b) or for MCP-1 (*P* = 0.0615; Fig. 1c). Additionally, the CM were also subjected to a prostaglandin E2 (PGE_2_) parameter assay; however, the PGE_2_ concentrations were not detectable (data not shown).

### aBMSCs induced immunosuppression on THP-1 monocytic cells

**Fig. 2:**
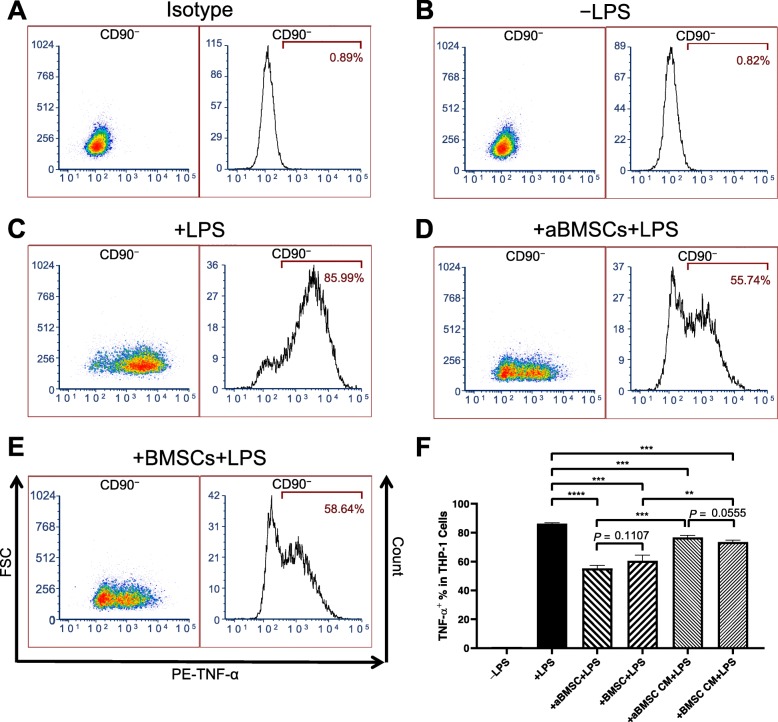
aBMSCs and BMSCs inhibited TNF-α expression in THP-1 cells in response to LPS stimulation. THP-1 cells were cultured in the presence or absence of aBMSCs or BMSCs or their CM for 72 h before treated with 1 μg/mL LPS and 5 μg/mL brefeldin A for 4 h. The non-adherent cells were collected and analyzed by flow cytometry using PerCP/Cy5.5-CD90 and PE-TNF-α antibodies. **a**–**e** Representative flow cytometric density plots and histograms of CD90^−^ cells (THP-1 cells). **a** Isotype control. **b** Naive THP-1 cells without LPS stimulation. (**c**) THP-1 cells cultured alone and treated with LPS. **d** THP-1 cells cocultured with aBMSCs and treated with LPS. **e** THP-1 cells cocultured with BMSCs and treated with LPS. **f** Quantification of TNF-α^+^ THP-1 cells in CD90^−^ population of indicated treatment groups. **P < 0.01. ***P < 0.001, ****P < 0.0001. *n* = 3 in each group, and aBMSCs and BMSCs were isolated from three different donors, respectively

Because MCP-1 has been implicated in the recruitment of monocytes and macrophages, we next initiated the investigation of aBMSCs’ immunomodulatory properties with coculture of aBMSCs and THP-1 human monocytic cells. Naive THP-1 cells without activation by LPS did not express the pro-inflammatory cytokine TNF-α (0.82 ± 0.01%; Fig. 2b, f). When THP-1 cells were stimulated with 1 μg/mL LPS for 4 h, an average of 86.30 ± 0.55% cells expressed TNF-α (Fig. 2c, f). In contrast, coculture with aBMSCs or BMSCs for 72 h significantly reduced the percentage of THP-1 cells expressing TNF-α in response to LPS stimulation to 55.25 ± 2.09% and 60.51 ± 3.94%, respectively (Fig. 2d, f). This suggests that aBMSCs has immunosuppressive effects on monocytes with at least the same potency as BMSCs. To determine whether the interaction between aBMSCs/BMSCs and THP-1 is attributed to the secretion of soluble factors, CM was incubated in the THP-1-stimulated cultures. The results showed that aBMSC CM and BMSC CM also suppressed the TNF-α expression in THP-1 cells, although to a lesser extent than cocultures of BMSCs and aBMSCs in direct contact (76.66 ± 1.53% and 73.50 ± 1.35%, respectively, Fig. 2f). This indicates that the soluble factors play an important role in the immunosuppressive functions of aBMSCs and BMSCs.

### aBMSCs skewed THP-1 macrophages into a highly phagocytic phenotype

**Fig. 3:**
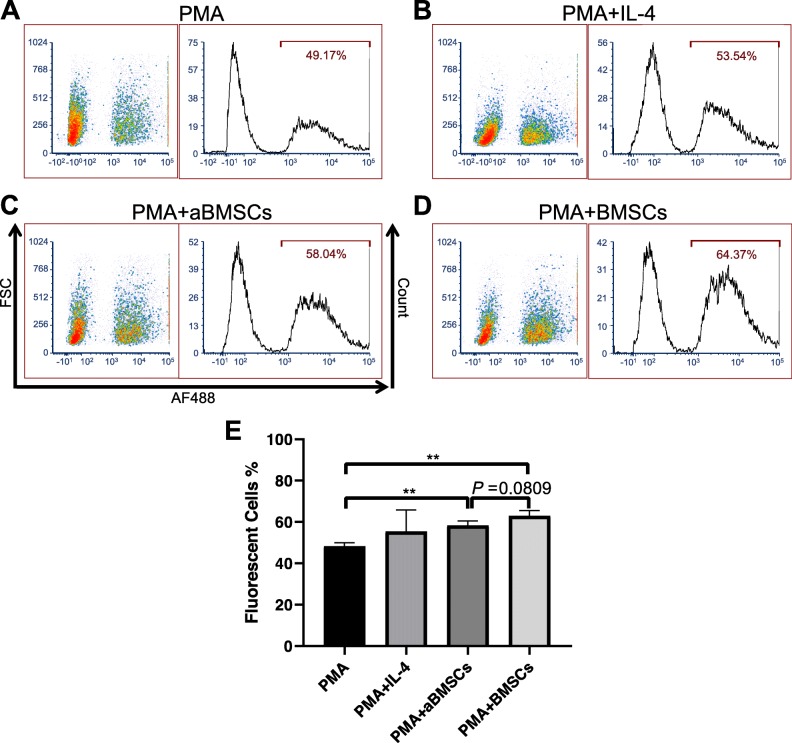
aBMSCs and BMSCs promoted phagocytosis of *E. coli* in THP-1 macrophages. THP-1 cells were incubated for 96 h with PMA to differentiate into macrophages in the presence or absence of aBMSCs/BMSCs cultured in Transwell inserts. THP-1 cells differentiated in the presence of IL-4 at 100 ng/mL for 72 h served as a M2 polarization control. THP-1 macrophages were treated with AF488-labeled *E. coli* for 1 h, followed by 1 min Trypan blue treatment to quench extracellular fluorescence. Cells were rinsed, detached, and subjected to flow cytometry to evaluate the phagocytic activity by counting AF 488-positive cells. **a**–**d** Representative flow cytometric density plots and histograms of THP-1 macrophages treated with PMA alone (**a**), PMA + IL-4 (**b**), PMA + aBMSCs (**c**), and PMA + BMSCs (**d**). **e** Quantitation of fluorescent THP-1 macrophages (AF488-positive events) in indicated treatment groups. **P < 0.01. *n* = 3 in each group, and aBMSCs and BMSCs were isolated from three different donors, respectively

One of the mechanisms involved in the immunosuppressive effects of MSCs is the induction of macrophages with immunomodulatory capacities, which greatly depend on the soluble factors secreted by MSCs; coculture of MSCs or MSC-CM can convert macrophages from M1 pro-inflammatory phenotype or resting status to an anti-inflammatory phenotype with low production of pro-inflammatory factors (e.g., TNF-α and IL-1β) and high expression of anti-inflammatory cytokines (e.g., IL-10) and phagocytic activities, which is similar to M2 phenotype prototypically induced by IL-4 [23, 25–27]. Thus, we differentiated THP-1 monocytic cells into macrophages by incubating them with 10 ng/mL phorbol 12-myristate 13-acetate (PMA) for 96 h in the presence or absence of aBMSCs or BMSCs cultured in Transwell inserts. After differentiation, the THP-1 macrophages were incubated with AF488-labeled *E. coli* for 1 h at 37 °C, and their phagocytic activity was assessed by the percentage of fluorescent cells among the total population. THP-1 macrophages cultured alone in PMA-containing medium had an average of 48.25 ± 1.71% cells that had undergone phagocytosis of *E. coli* (Fig. 3a, e). Both aBMSCs and BMSCs, even without direct cell-cell contact, significantly increased the phagocytic activity of THP-1 macrophages to 58.30 ± 2.29% and 62.92 ± 2.58%, respectively, which were comparable to THP-1 macrophages differentiated in the presence of IL-4 at 55.41 ± 10.34% (Fig. 3b–e). The difference between aBMSC- and BMSC-treated groups was not statistically significant (*P* = 0.0809; Fig. 3e), indicating that aBMSCs as well as BMSCs skewed monocytes to differentiate into anti-inflammatory macrophages with higher phagocytic activities.

### aBMSCs inhibited T cell activation and proliferation

**Fig. 4:**
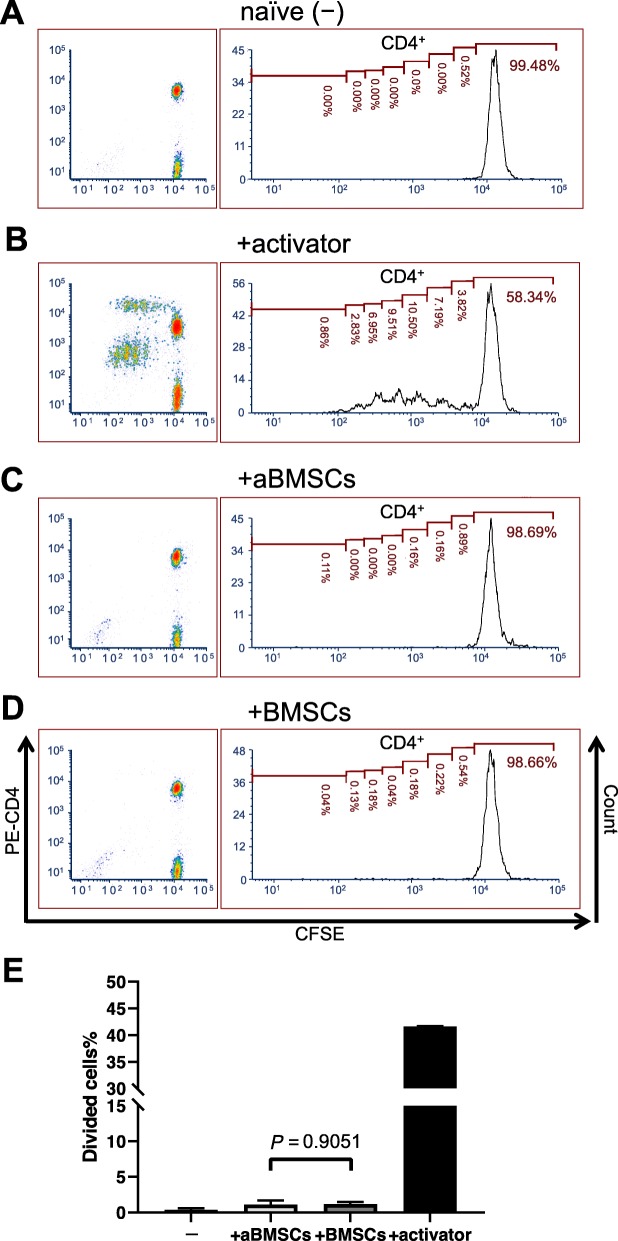
aBMSC and BMSCs did not evoke T cell proliferation. Primary PBMCs prelabeled with CFSE (1 × 10^5^) were cultured in the presence or absence of aBMSCs or BMSCs (1 × 10^4^) with or without a T cell activator for 5 days. Non-adherent cells were harvested and analyzed by flow cytometry using PE-CD4 antibody to label T cells. **a**–**d** Representative flow cytometric density plots of PBMCs and histograms of CD4^+^ T cells. **a** Naïve PBMCs cultured alone without activator. **b** PBMCs cocultured with BMSCs without activator. **c** PBMCs cocultured with aBMSCs without activator. **d** PBMCs cultured alone with activator. **e** The percentage of divided CD4^+^ T cells at day 5 of indicated treatment groups. *n* = 2 for PBMCs cultured alone with or without activator, *n* = 6 for PBMCs cocultured with aBMSCs from three different donors, and *n* = 3 for PBMCs cocultured with BMSCs from three different donors

**Fig. 5:**
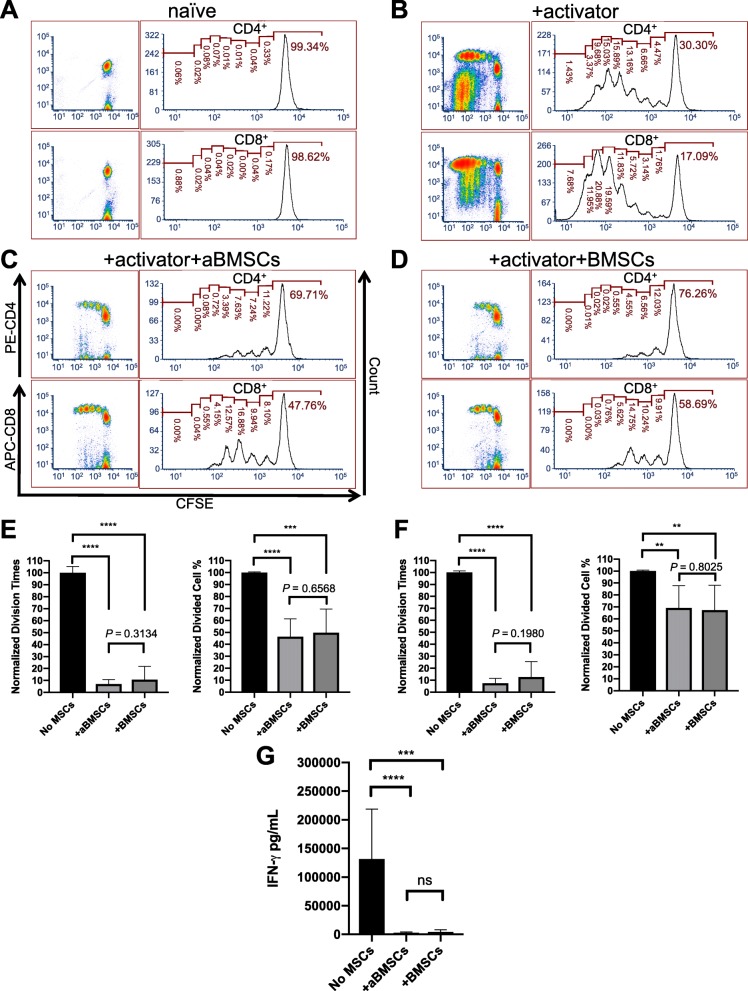
aBMSC and BMSCs inhibited T cell activation and proliferation. Primary PBMCs prelabeled with CFSE (2 × 10^5^) were cultured in the presence or absence of aBMSCs or BMSCs (1 × 10^4^) with or without a T cell activator for 5 days. Non-adherent cells were harvested and analyzed by flow cytometry using PE-CD4 and APC-CD8 antibodies to label T cells. **a**–**d** Representative flow cytometric density plots of PBMCs and histograms of CD4^+^ and CD8^+^ T cells. **a** Naïve PBMCs cultured alone without activator (negative control). **b** PBMCs cultured alone with activator (positive control). **c** PBMCs cultured with aBMSCs and activator. **d** PBMCs cultured with BMSCs and activator. **e**–**f** The division times and the percentage of divided cells in CD4^+^ (**e**) and CD8^+^ T cells (**f**) at day 5 normalized according to the positive and negative controls. **g** The IFN-γ levels in the culture medium at day 5 in the indicated treatment groups determined by ELISA. **P < 0.01. ***P < 0.001. ****P < 0.0001. *n* = 4 for PBMC cultured alone with activator, *n* = 12 for PBMCs cocultured with aBMSCs from three different donors, and *n* = 11 for PBMCs cocultured with BMSCs from three different donors

To determine if aBMSCs affect T cell-mediated adaptive immunity, we cocultured them with PBMCs containing T cells and evaluated their effects on T cell activation and proliferation. Without the use of T cell activator, T cells in the PBMCs cultured alone did not proliferate (Figs. 4a and 5a). We first confirmed that aBMSCs as well as BMSCs did not evoke activation and proliferation of analogous T cells with the presence of antigen-presenting cells in PBMCs: Only 1.123 ± 0.555% in CD4^+^ T cells were divided cells after 5 days of coculture with aBMSCs, which was similar to 1.167 ± 0.300% in CD4^+^ T cells in BMSC coculture (Fig. 4). Thus, aBMSCs appear to be immunoevasive as BMSCs have been shown to be. When the anti-CD3/CD28/CD2 activator was added, it mimicked in vivo activation from antigen-presenting cells, and T cells underwent a series of cell divisions (Figs. 4b and 5b). However, in response to the activator at day 5, both aBMSCs and BMSCs significantly diminished the CD4^+^ T cell division times to 6.42 ± 3.73% and 7.07 ± 5.99%, respectively, compared to the stimulated PBMC monoculture (Fig. 5e). The percentage of divided cells in CD4^+^ population at day 5 was reduced to 46.55 ± 14.81% by aBMSCs and 44.92 ± 17.85% by BMSCs compared to the monoculture (Fig. 5e). Similar immunosuppressive effects were observed in CD8^+^ T cells where the number of divisions was only 7.56 ± 4.07% or 8.70 ± 8.41% in the presence of aBMSCs or BMSCs, respectively, and the divided CD8^+^ cells were 69.18% ± 18.55% and 63.02% ± 19.96%, respectively (Fig. 5f). Again, there was no difference between aBMSC- and BMSC-cocultured groups (Fig. 5e). To further assess the activation of T cells, we collected their culture supernatant and measured the IFN-γ levels by ELISA. The IFN-γ results were in agreement with the reduced proliferation rates seen in the cocultures, whereby both aBMSCs and BMSCs substantially reduced T cell secretion of IFN-γ to a similar degree (Fig. 5f).

## Discussion

The recently identified immunomodulatory properties of MSCs have generated high interest for the therapeutic use of MSCs to treat chronic inflammatory conditions. While preclinical studies conducted in murine models have predominantly positive results, the outcomes of clinical trials in human subjects are mixed [17]. In fact, the first major industry-sponsored phase III trial of MSCs (Prochymal) to treat steroid-refractory GvHD concluded that these allogeneic BMSCs did not significantly enhance the overall response rate comparing to placebo [17]. Nonetheless, Prochymal showed benefit in pediatric patients [28] and received approval to treat children with acute GvHD in Canada. In addition, BMSC injection has become an acceptable treatment for acute steroid-refractory GvHD in the UK and Japan [17]. However, debates on the effectiveness continue, which calls for a deeper understanding of the mechanisms. Keto et al. reported in 2018 that the lymphocyte profiles of responders and non-responders were similar among 16 acute GVHD patients’ outcomes after the treatment of third-party BMSCs [29]. Relative to MSCs derived from the bone marrow of the iliac crest (BMSCs) that are the most intensively investigated in the preclinical and clinical studies of MSC therapies, alveolar bone-derived MSCs (aBMSCs) are a more readily accessible and abundant tissue source of MSCs yet their immunomodulatory properties have not been previously described. The aim of this study was to characterize the immunomodulatory properties of aBMSCs relative to BMSCs through cytokine production as well as their influence on different immune cells.

In this study, we first determined that similar to BMSCs, aBMSCs cultured alone without stimulation do not secrete inflammatory cytokines and chemokines except IL-6 and MCP-1 (CCL2), yet at slightly lower levels. Additionally, unstimulated aBMSCs do not secrete high levels of PGE_2_, which was similar to unstimulated BMSCs as we and others have observed [30]. IL-6 is a pleiotropic cytokine with context-dependent pro- and anti-inflammatory properties [31]. In the classic acute inflammatory episode, neutrophils are first recruited to the inflammatory site, followed by monocytes and lymphocyte infiltration to replace neutrophils, and subsequent tissue repair. IL-6 is an indispensable cytokine for a normal neutrophil generation (granulopoiesis) and function (respiratory burst and degranulation) [31, 32] and also regulates neutrophil accumulation by modulating the expression of chemokines and adhesion molecules in stromal tissue cells [31, 33]. In regard to monocytes, IL-6 drives monocytes differentiation towards macrophages rather than dendritic cells (DCs) [34, 35], favoring anti-inflammatory phenotypes associated with wound healing, reduced microbicidal activities, secretion of pro-inflammatory cytokines, and enhanced expression of the M2 macrophage marker CD206 [31, 36]. IL-6 also plays key roles in the regulation of lymphocytes including roles in survival, expansion, and maturation of B cells and plasmablasts, and the T cell proliferation, survival, differentiation, and cytokine expression [31].

MSC production of MCP-1 is also of high interest in that one of its major roles is to recruit monocytes as well as macrophages [37], but it also affects monocyte activation and macrophage polarization. The transition of macrophages from M1 pro-inflammatory to M2 anti-inflammatory phenotype is key to the resolution of inflammation and tissue restoration [38]. MCP-1 has been shown to induce M2 polarization of macrophages and promote IL-10 secretion in response to LPS by macrophages differentiated by GM-CSF in vitro [36, 39]. In a mouse model of foreign body response, wild type macrophages was found to undergo unique polarization that expresses both M1 and M2 markers, whereas MCP-1 knockout macrophages were defected in fusion and formation of foreign body giant cells, and the induction of TNF-α and activation of the canonical NF-κB pathway were compromised [40, 41]. Yet, some studies using transgenic mice with systemic or tissue-specific expression of MCP-1 suggest that MCP-1 recruits circulating monocytes at a low level, which then differentiate into different phenotypes of macrophages according to other mediators in loco [42]. Additionally, DCs, Langerhans cells, and T and B lymphocytes have also been found to be infiltrated in MCP-1 transgenic mice [42]. Furthermore, MCP-1 is also involved in Th2 polarization and enhances the secretion of IL-4 by T cells [37, 43]. Interestingly, there is a discrepancy in previous studies in regard to the migration of BMSCs to MCP-1. Some groups have shown that MCP-1 is chemotactic to human and rodent BMSCs [44–48], and its receptor CCR2 was found to be expressed in human and rat BMSCs [45, 48]. This may contribute to the recruitment of BMSCs to inflammatory sites in vivo. However, Ringe et al. reported that human BMSCs did not migrate towards MCP-1 [49] and Takano et al. reported similar findings with rat BMSCs [50].

Both IL-6 and MCP-1 play key roles in regulating monocyte/macrophage phenotype and activities. BMSC secretion of IL-6 has been reported to skew monocyte differentiation from CD14^−^CD1a^+^ DCs to CD14^+^CD1a^−^ cells (which have a lower immunostimulatory capacity towards anti-inflammatory macrophage differentiation) [51]. Using mouse macrophages lacking α chain of IL-6 receptor, Philipp et al. suggested that IL-6 signaling is indispensable for alternative activation of macrophages by BMSC coculture or IL-4 treatment [52]. Therefore, we hypothesized that aBMSCs have immunomodulatory effects on monocytes and macrophages similar to BMSCs. Cocultures of THP-1 monocytic cells or macrophages with aBMSCs or BMSCs were carried out and the phenotypes following coculture in response to inflammatory stimuli were evaluated. When stimulated by LPS, THP-1 cells turned into a pro-inflammatory phenotype with high levels of TNF-α expression. When co-cultured with either aBMSCs or BMSCs, LPS-induced TNF-α expression in THP-1 cells was significantly inhibited. This effect is at least partially due to their secretion of soluble factors, because the TNF-α-expressing THP-1 percentages were also reduced by the aBMSC or BMSC CM without contacting MSCs. Furthermore, we also have shown that aBMSCs and BMSCs, even without cell-to-cell contact, significantly increased the phagocytic activities of THP-1 macrophages like IL-4, a classic inducer of M2 polarization [27]. This finding suggests that their ability to skew macrophage differentiation into an anti-inflammatory phenotype is at least in part dependent on the secretion of soluble factors. This is in alignment with a number of other studies that have demonstrated that BMSCs and MSCs from other tissue origins induce monocyte differentiation into anti-inflammatory macrophages [22, 23, 25, 26, 53].

It is well established that macrophages not only play powerful roles in innate immunity but their M1/M2 phenotypes also generally cause Th1/Th2 responses, respectively, in adaptive immunity, thereby implicating a potentially important role for them in autoimmunity as well [38]. T lymphocytes play a central role in cell-mediated adaptive immunity, which becomes malignant in patients who received allografts and those of autoimmune diseases such as type I diabetes, multiple sclerosis, rheumatoid arthritis, and SLE. Many studies have confirmed varieties of MSCs including BMSCs inhibit T cell activation and proliferation [24, 54–57]. To evaluate the immunomodulatory functions of aBMSCs in adaptive immune responses, we used a well-established T cell activation and proliferation model where the activator comprised of antibodies that bind CD3 and CD28 surface ligands provides the primary and costimulatory signals required for T cell activation. Considering that monocytes seem to be indispensable for the suppression of T cell proliferation by BMSCs and MSCs from the umbilical cord, umbilical cord blood [55], and placenta [56], we used primary human PBMCs consist of lymphocytes (T cells, B cells, nature killer cells) and monocytes instead of purified T cells. Our results showed that aBMSCs cocultured with PBMCs even at a 1:20 ratio had substantial immunosuppressive effects on T cell responses to the same extent as BMSCs. Prior evidence has indicated that the BMSCs utilize different mechanisms to suppress T lymphocyte proliferation in response to mitogens or alloantigens in vitro: suppression on phytohemagglutinin-induced T cell proliferation was partially attributed to PGE_2_ released by BMSCs whereas in mixed (allogeneic) lymphocyte culture BMSCs increased levels of IL-10, IL-2, and soluble IL-2 receptor [57]. PGE_2_ production was also attributed to the monocyte-dependent suppression on mitogen-induced T cell proliferation by umbilical cord MSCs [55]. Also, different types of MSCs may use different mechanisms to modulate T cell responses [56, 58]. Finally, the favor of monocytes for M2 macrophage differentiation rather than DC differentiation is considered as an indirect suppression on T cell response [53]. Further studies are required to reveal which mechanisms aBMSCs use to block T cell proliferation in different scenarios.

IFN-γ is a pivotal pro-inflammatory cytokine, and in the current study, we also confirmed that both BMSCs and aBMSCs can inhibit IFN-γ production by cultured PBMCs. According to Ren et al., the initial production of IFN-γ is required for the immunosuppressive effects of BMSCs, because mouse BMSCs lacking IFN-γ receptor 1 failed to suppress anti-CD3-activated splenocyte proliferation in coculture [59]. Yet, anti-CD3-activated T cells were found to induce BMSC apoptosis through the Fas/Fas ligand pathway in mouse (tested in 5:1 coculture) [60]. Similar cell death was also observed in the mouse orofacial bone/bone marrow-derived MSCs cocultured with pan T cells activated by anti-CD3 antibody [61]. Furthermore, Liu et al. found that pro-inflammatory T cells inhibit the osteogenesis of BMSCs by secreting IFN-γ, which upregulated Smad6 expression in BMSCs [62]. However, treatment of IFN-γ, ranging from 10 to 200 ng/mL, did not induce mouse BMSC apoptosis under their experiment settings in spite of the IFN-γ-induced upregulation of Fas expression [62]. Nonetheless, they found that IFN-γ enhance TNF-α-induced BMSC apoptosis, and the combination of TNF-α and IFN-γ induced Fas internalization and clustering in a Fas ligand-independent manner and selectively inhibited TNF receptor 2-mediated anti-apoptotic effect [62].

In the current study, we have confirmed that aBMSCs are immunoevasive in vitro for T cell proliferation was barely observed when cocultured with allogeneic PBMCs, and they have immunomodulatory properties comparable to BMSCs. A number of animal models have demonstrated that following injections of BMSCs to treat different inflammatory conditions (GvHD, bisphosphonate-related osteonecrosis of the jaw, allogeneic skin graft, etc.), and immunomodulatory activities of BMSCs were responsible for successful outcomes of the cell therapy approaches [63–65]. Furthermore, the infusion of MSCs has been implied in clinical trials to treat GvHD [18, 19], SLE [66], rheumatoid arthritis [67], multiple sclerosis, and amyotrophic lateral sclerosis [68, 69]. Considering that the alveolar bone tissue is a more accessible and cost-effective source of MSCs compared to the iliac crest bone marrow, aBMSCs can be a better alternative to BMSCs for immunomodulatory cell therapies although preclinical studies are needed to further evaluate these promising findings.

## Conclusions

In summary, this study characterizes the immunomodulatory properties of aBMSCs in comparison with BMSCs, both of which have potent effects on immune cells. aBMSCs induce a less inflammatory monocyte phenotype and a more anti-inflammatory macrophage phenotype and significantly inhibit T cell activation and proliferation. The secretome of aBMSCs and BMSCs contribute to their immunosuppressive functions, with MCP-1 and IL-6 being two inflammatory cytokines at high levels. Taken together, aBMSCs should be considered a viable stem cell candidate for immunomodulatory cell therapies aimed to treat inflammatory conditions.

## Data Availability

The data that support the findings of this study are available from the corresponding author upon reasonable request.

## Abbreviations

aBMSCs: Alveolar bone-derived mesenchymal stem cells
BMSCs: Bone marrow-derived mesenchymal stem cells
CCL2: C-C motif chemokine ligand 2
CCR2: C-C motif chemokine receptor 2
CFSE: 5-(and 6)-Carboxyfluorescein diacetate succinimidyl ester
CM: Conditioned medium
DCs: Dendritic cells
*E. coli*: *Escherichia coli*
ELISA: Enzyme-linked immunosorbent assay
GvHD: Graft versus host disease
IFN-γ: Interferon γ
IL: Interleukin
LPS: Lipopolysaccharide
MCP-1: Monocyte chemoattractant protein 1
MSCs: Mesenchymal stem cells
PBMCs: Peripheral blood mononuclear cells
PGE_2_: Prostaglandin E2
PMA: Phorbol 12-myristate 13-acetate
SLE: Systemic lupus erythematous
TNF-α: Tumor necrosis factor α
